# Symbiont dynamics of the Tibetan tick *Haemaphysalis tibetensis* (Acari: Ixodidae)

**DOI:** 10.1186/s13071-017-2199-0

**Published:** 2017-05-25

**Authors:** Rongrong Wang, Ningxin Li, Jiannan Liu, Tuo Li, Ming Liu, Zhijun Yu, Jingze Liu

**Affiliations:** 1 Key Laboratory of Animal Physiology, Biochemistry and Molecular Biology of Hebei Province, College of Life Sciences, Hebei Normal University, Shijiazhuang, 050024 China; 2 Institute of Biochemistry, Department of Biology, Carleton University, 1125 Colonel By Drive, Ottawa, ON K1S 5B6 Canada

**Keywords:** *Haemaphysalis tibetensis*, Endosymbionts, *Coxiella*, *Rickettsia*, Tissue distribution, Population dynamics

## Abstract

**Background:**

Characterization of the microbial diversity and symbiont dynamics of ticks may help to understand the development of ticks and reveal new strategies to control tick-transmitted pathogens, which has not yet been explored in the Tibetan tick *Haemaphysalis tibetensis*. This tick species is widely distributed in the Tibetan Plateau, and is recognized as one of the primary parasites affecting domestic and wild animals.

**Methods:**

In the present study, the endosymbionts of *H. tibetensis* were characterized using diagnostic polymerase chain reaction (diagnostic PCR), and further evaluated for tissue distribution and population dynamics at each developmental stage of ticks and in tissues at different reproductive statuses by real-time quantitative polymerase chain reaction (RT-qPCR).

**Results:**

Two symbionts were found in *H. tibetensis*, and named as CLS-Ht (*Coxiella-*like symbiont in *H. tibetensis*) and RLS-Ht (*Rickettsia-*like symbiont in *H. tibetensis*). They showed 100% infection rate in both females and males of *H. tibetensis*. CLS-Ht and RLS-Ht can be observed within eggs, larvae, nymphs and adults, which indicates vertical transmission in *H. tibetensis*. CLS-Ht was specifically distributed in the female ovaries and Malpighian tubules, whereas RLS-Ht was detected within ovaries, Malpighian tubules, salivary glands and midguts of the ticks. Real-time qPCR suggested that adult ticks carried the largest amount of CLS-Ht and RLS-Ht with CLS-Ht having a significantly higher presence in females than in males (*P* < 0.05), whereas the presence of RLS-Ht showed no significant differences between sexes. In the ovaries, CLS-Ht distribution reached a peak at one day post-engorgement, and then gradually declined to a lower level, whereas no change was observed in RLS-Ht. In Malpighian tubules, the amount of both symbionts displayed an increasing trend with time post-engorgement. In midguts and salivary glands, the amount of RLS-Ht showed no significant differences.

**Conclusion:**

Two novel endosymbionts (CLS-Ht and RLS-Ht) were characterized in *H. tibetensis* both showing a high prevalence and stable vertical transmission. The described tissue distribution and population dynamics might imply the important functions of these symbionts during the development and reproduction of ticks.

**Electronic supplementary material:**

The online version of this article (doi:10.1186/s13071-017-2199-0) contains supplementary material, which is available to authorized users.

## Background

Ticks are obligate blood-sucking ectoparasites of many vertebrate animals and can transmit a diversity of pathogens including bacteria (rickettsiae and spirochetes), viruses and protozoans [1]. As the worldwide distribution and dynamic frequency from ‘on-host’ to ‘off-host’ changes, the involvement of ticks in commensal, mutualistic or parasitic interactions with different kinds of microorganisms becomes unavoidable [2, 3].

Many symbionts and complex bacterial communities have been explored from different tick species [4, 5], different tissues and organs [6, 7], different life stages [8] and different feeding statuses [9]. These symbionts include *Coxiella*-like symbionts [10], *Rickettsia*-like symbionts, *Arsenophonus*-like symbionts [11], *Francisella*-like symbionts [12, 13], “*Candidatus* Midichloria mitochondrii” and *Wolbachia*-like symbionts [14]. Some microbes have been shown to provide the necessary nutrition needed for tick development whereas others have been shown to interfere with survival and transmission of tick-borne pathogens [15]. Among the tick-associated symbionts, *Coxiella*-like symbionts have been detected in several genera of ticks [16, 17], and were found mainly infecting ovaries and vertically transmitted by transovarial transmission [18, 19]. Eliminating of *Coxiella*-like symbionts with antibiotics could cause severe reduction in fecundity and fitness of *Amblyomma americanum* [20], and recent genome studies on *Coxiella*-like symbionts in *A. americanum* and *Rhipicephalus turanicus* suggested their specific functions in providing required nutrients lacking in a blood meal [21, 22]. Furthermore, *Coxiella*-like symbionts could impact the colonisation and transmission of other pathogens [16]. Similarly, *Rickettsia*-like symbionts characterized in *Dermacentor variabilis* and *Dermacentor andersoni* show little or no pathogenicity in laboratory animials, but can influence the physiology of host ticks and affect the transmission of the coinfected pathogenic rickettsiae [16]. A metabolic reconstruction on the genome of *Rickettsia* endosymbionts in both *Ixodes scapularis* and *Ixodes pacificus* has revealed the present of folate (B9 vitamin) biosynthesis genes [23]. Hence, characterizing the symbiont dynamics in ticks may help understand the development of ticks and reveal new strategies to control tick-transmitted pathogens.

The tick *Haemaphysalis tibetensis* is an important endemic-pathogen vector in the Qinghai-Tibet Plateau, from which new strains of spirochete and orbivirus were isolated [24, 25]. This species of tick can occur above an altitude of 4,000 m where the environment is cold and dry [26]. The microbial diversity and symbiontic dynamics of *H. tibetensis* have not yet been explored, therefore, the current study investigated the endosymbionts in *H. tibetensis*, and the tissue tropism, population dynamics and vertical transmission of these endosymbionts were further evaluated in the hope of a better understanding the relationship between this species of tick and its different microorganisms.

## Methods

### Collection and rearing of ticks

Free-living *H. tibetensis* ticks were collected by flag dragging from vegetations in the Damxung County (90°45′–91°31′E, 29°31′–31°04′N; altitude 4,353 m), north Lhasa City, Tibet Autonomous Region, China, and identified according to available characteristics [27–29]. Part of the collected ticks (defined as field colony) were frozen in liquid nitrogen and then preserved at -80 °C until use; others were reared on domestic rabbits *Oryctolagus cuniculus* as described previously [30]. Offspring of *H. tibetensis* (defined as laboratory colony) were maintained at 26 °C, humidity 80% with a light: dark regime of 16:8 h.

### Dissection of ticks

Ticks were first surface sterilized with 70% ethanol (3 washes) and then dissected sterilely under a stereomicroscope at 10 × 23 magnification using a micro-clipper in sterile phosphate-buffered saline (PBS) (137 mM NaCl, 2.7 mM KCl, 4.3 mM Na_2_HPO_4_ · 7H_2_O, 1.4 mM KH_2_PO_4_, pH 7.4) as described previously [31]. Specific organs including ovaries, Malpighian tubules, salivary glands and midguts were separately collected in 1.5 ml sterile vials (Axygen, Union City, USA) and frozen in -80 °C for subsequent use.

### Genomic DNA extraction

Total genomic DNA was extracted from each group of adults (10 females and 10 males, respectively) from field or laboratory colonies, and from dissected tissues and organs using a Genomic DNA isolation kit (Qiagen, Hilden, Germany). The concentration of the extracted genomic DNA was measured using a Nanodrop (Thermo Fisher Scientific, Waltham, USA) and the purity was evaluated by electrophoresis of the extracts in 1% (w/v) agarose gels.

### Bacterial 16S rRNA gene library and restriction fragment length polymorphism (RFLP) analysis

The bacterial 16S rRNA gene library was constructed using the genomic DNA of pooled ticks from field and laboratory colony. A ~1,500 bp fragment of 16S rRNA gene was amplified using bacterial universal primers Eub27F/Eub1492R [32] (Table 1). Resultant PCR products were purified with a PCR Purification Kit (Bioteke, Beijing, China) and ligated into the pEASY-T1 cloning vector using the pEASY-T1 simple cloning kit (TransGen, Beijing, China). Recombinant DNA was transformed into *Escherichia coli* TOP10 competent cells (TransGen, China). Thereafter, both *Hae III* and *RsaI* restriction endonucleases were applied to digest the gene library for subsequent RFLP analysis. Positive clones with different restriction fragment patterns were sequenced (Sangon Biotech, Shanghai, China) and blasted in the NCBI database (http://www.ncbi.nlm.nih.gov/BLAST/).

**Table 1:** Oligonucleotide primers used for PCR amplification and sequencing

Primer	Species	Target gene	Nucleotide sequence (5′–3′)	Annealing temperature (°C)	Approx. product size (bp)	Reference
CLS-F	*Coxiella*	16S rRNA	CACGTAGGAATCTACCTTGTAG	55	90	[4]
CLS-R			CGTTTTGTTCCGAAGAAATTAT			
Eub27F	Eubacteria	16S rRNA	AGAGTTTGATCCTGGCTCAG	55	1,500	[32]
Eub1492R			TACCTTGTTACGACTT			
Rickettsia354F	*Rickettsia*	16S rRNA	CAGCAATACCGAGTGAGTGATGAAG	56	350	[33]
Rickettsia647R			AGCGTCAGTTGTAGCCCAGATG			
Actin-F	*Haemaphysalis*	actin	CGTTCCTGGGTATGGAATCG	55	100	[4]
Actin-R	*Haemaphysalis*	actin	TCCACGTCGCACTTCATGAT			

### Phylogenetic analysis

The 1,500 bp 16S rRNA gene sequences obtained were compared with known sequences listed in the GenBank nucleotide sequence databases, aligned using the CLUSTAL W program, and manually checked with excluded DNA gaps. The phylogenetic trees were produced according to the neighbor-joining method after Kimura 2-parameter correction in the MEGA version 6 using bootstrap analyses with 1,000 replicates; the gram-positive bacterium *Bacillus subtilis* (X60646) was used as the outgroup species.

### Prevalence, tissue distribution and dynamic of the symbionts

The prevalence, vertical transmission and dynamics of the symbionts were evaluated and monitored using SYBR green-based real-time qPCR. Briefly, genomic DNA was isolated from several groups including individual adults (50 females and 50 males) from the laboratory colony, and pooled samples of ticks in various developmental stages (500 eggs, 200 larvae and 50 nymphs, respectively), or from different organs. Standard curves were established by serial dilutions of plasmids containing inserts of the amplified 16S rRNA gene sequences from symbionts and host (tick) actins (Table 1). The 25 μl master mix was composed of 12.5 μl of 2× TransStart^TM^ Top Green qPCR SuperMix (TransGen, China), 0.5 μl of each 10 μM primer [33] (Table 1), 10.5 μl H_2_O and 1 μl template DNA. The qPCR assays were conducted in 96-well polypropylene plates in a Mx3005P qPCR system (Agilent Technologies, Santa Clara, USA) and conditions were set as follows: 94 °C for 30 s; 40 cycles of 94 °C for 5 s and 60 °C for 30 s. The primers with high amplification specificity were verified by unique peaks observed in corresponding melting curves. Each plate contained triplicate reactions for each DNA sample. Melting curves were also traced after each assay to confirm that the fluorescence signal had been retrieved from specific PCR products and to ensure the absence of primer dimers. Sterile water was used as the negative control. Parametric data were tested by t-tests and one-way analysis of variance using SPSS 17.0 for Windows software (SPSS Inc, Chicago, USA).

## Results

### Identification and phylogenetic analysis of symbionts

**Fig. 1:**
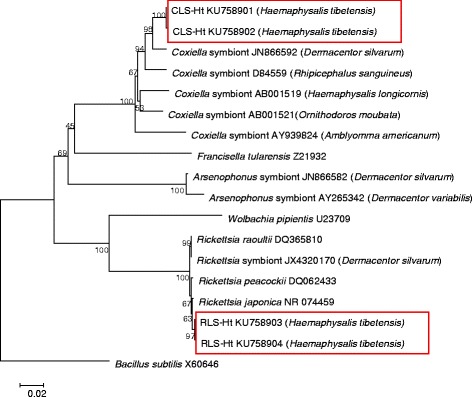
Neighbor-Joining unrooted phylogenetic tree of CLS-Ht and RLS-Ht symbionts of *H. tibetensis* and other tick-associated symbionts. The percentage of replicate trees in which the associated taxa clustered together in the bootstrap test (1,000 replicates) is shown next to the branches. Genetic distance was computed using the Kimura 2-parameter method and are in the units of the number of base substitutions per site. Sequence alignments and tree generation were conducted in MEGA6

After constructing 16S rRNA gene libraries and RFLP analyses, two different bacterial genera were detected from the *H. tibetensis*. After submitting the sequences to GenBank, about 99 and 97% of the sequences showed sequence similarities with *Rickettsia japonica* (GenBank NR074459) and *Coxiella*-like symbionts from *Dermacentor silvarum* (GenBank JN866592), and therefore they were assigned into genera *Coxiella* (GenBank KU758901 and KU758902) and *Rickettsia* (GenBank KU758903 and KU758904) and ultimately named as CLS-Ht (*Coxiella-*like symbiont in *H. tibetensis*) and RLS-Ht (*Rickettsia-*like symbiont in *H. tibetensis*). After phylogenetic analysis, the partial 16S rRNA gene sequence of CLS-Ht proved to be close to that of the symbiotic *Coxiella* in *Rhipicephalus sanguineus* (GenBank D84559), and RLS-Ht clustered with *Rickettsia peacockii* (GenBank DQ062433) (Fig. 1).

### Prevalence of symbionts

A total of 20 females and 20 males from the field colony were collected and studied by diagnostic PCR and sequencing. The results showed that all the ticks were CLS-Ht and RLS-Ht positive, suggesting that the infection rate of CLS-Ht and RLS-Ht might be 100% in *H. tibetensis* (Additional file 1: Figure S1).

### Vertical transmission of the symbionts

To test whether transmission of CLS-Ht and RLS-Ht was transovarial or transstadial, samples of eggs, the first generation (F1) larvae, F1 nymphs, F1 females and F1 males were screened. All the tick extracts were infected with CLS-Ht and RLS-Ht, which is consistent with vertical transmission (Additional file 2: Figure S2).

### Tissue distribution of the symbionts

The distribution analysis revealed that CLS-Ht was sepcifically harbored in ovaries and Malpighian tubules, whereas RLS-Ht was harbored in ovaries, Malpighian tubules, salivary glands and midguts of *H. tibetensis* (Additional file 3: Figure S3).

### Population dynamics of the symbionts

**Fig. 2:**
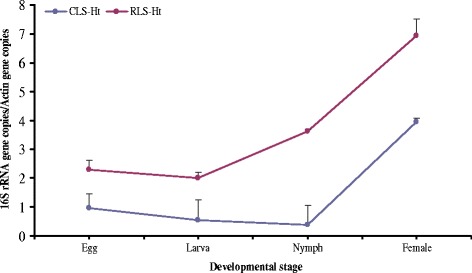
The density dynamics of CLS-Ht and RLS-Ht with *H. tibetensis* at different developmental stages. Means and standard errors of means are shown

**Fig. 3:**
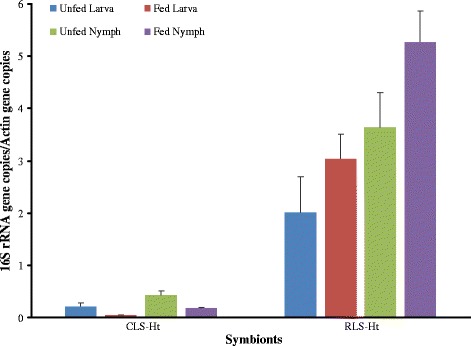
The quantitative dynamic of CLS-Ht and RLS-Ht in *H. tibetensis* before and after feeding. Means and standard errors of means are shown

**Fig. 4:**
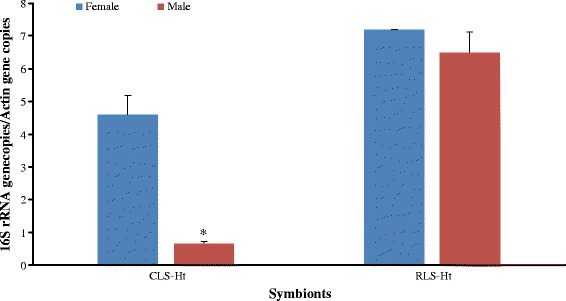
The quantitative dynamic of CLS-Ht and RLS-Ht in female and male *H. tibetensis*. Means and standard errors of means are shown, and asterisk indicates a statistical difference in each group (*P* < 0.05)

The density of CLS-Ht was high in adults but was at a low level in eggs, larvae and nymphs. For RLS-Ht, the results showed a low level in eggs and larvae but an increasing trend was seen from larvae to nymphs, and reached the peak in adults (Figs. 2 and 3). After feeding of larvae and nymphs, the CLS-Ht was decreased, whereas the RLS-Ht was increased (Fig. 3). When compared between sexes, CLS-Ht abundance was significantly higher in females than in males (*t*_(4)_ = 5.43, *P* = 0.011), whereas no obvious differences were observed in RLS-Ht between females and males (*t*_(4)_ = 0.92, *P* = 0.41) (Fig. 4).

**Fig. 5:**
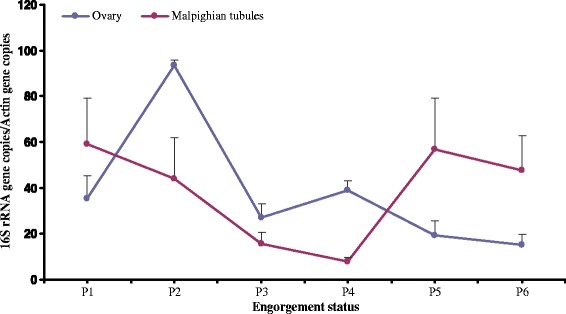
The dynamics of CLS-Ht in the ovaries and the Malpighian tubules of *H. tibetensis*. P1–6: 1–6 days post-engorgement. Means and standard errors of means are shown

In the ovaries, the densities of CLS-Ht were at lower levels in non-engorged stages, and increased on the first day after engorgement but subsequently declined slightly on the 5^th^ day after engorgement (Fig. 5). In Malpighian tubules, CLS-Ht was high one day after engorgement, declined the second day after engorgement, and thereafter the CLS-Ht increased again until the fifth day after engorgement.

**Fig. 6:**
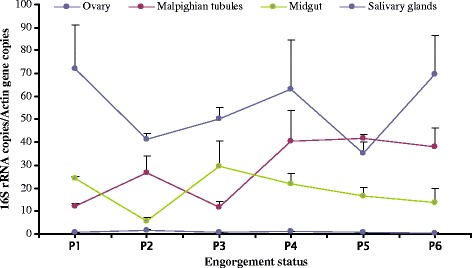
The dynamics of RLS-Ht in different tissues of *H. tibetensis*. P1–6: 1–6 days post-engorgement. Means and standard errors of means are shown

The amount of RLS-Ht in ovaries of *H. tibetensis* was significantly higher than in any other organ (*F*_(4, 21)_ = 7.19, *P* = 0.012), where the highest copy ratio reached 70 and varied randomly with time. In Malphibian tubules, the amount of RLS-Ht showed an elevating trend with copy ratio increasing from 10 to 40. In midgut, no obvious changes occurred, as the lowest copy ratio seen in the first day of engorgement (which was below 10) remained fairly stable afterward at 20. In the salivary glands, there were no obvious changes with copy ratio consistently below 2 (Fig. 6).

## Discussion

Ticks are notorious for acting as vectors and also serve as reservoirs of a great diversity of mammalian pathogens, hence the microbiome and endosymbionts within ticks have attracted the attention of researchers since each tick species harbors its own unique bacterial community [3, 34]. In the Tibetan tick *H. tibetensis*, CLS-Ht and RLS-Ht were characterized by constructing 16S rRNA libraries and RLFPs. Both CLS-Ht and RLS-Ht showed 100% infectivity and vertical transmission in *H. tibetensis*; however, differences in tissue-specific distribution was observed. The CLS-Ht mainly infects the ovaries and the Malpighian tubes, which is consistent with the previous observation of *Coxiella*-like symbionts in *R. sanguineus* and *R. turanicus* ticks [18]. No tissue-specific infectivity was observed for RLS-Ht, a conclusion that is similar with the *Rickettsia-*like symbiont distribution in *H. longicornis* and *D. silvarum* found previously [4]. The coinfection of symbionts is common in ticks. Endosymbionts belonging to the genera *Rickettsia*, *Coxiella* and *Arsenophous* have been found in *A. americaum* where species of all of the three genera showed vertical transmission [35]. The *Coxiella*-like and *Francisella*-like symbionts were found coinfected in *Ornithodorus moubata* [36, 37]. In the tick *R. turanicus* and *R. sanguineus*, both *Coxiella*-like and *Rickettsia*-like symbionts were detected, and densities were overall stable throughout the questing season [38].

In the tick *H. tibetensis*, the density of both CLS-Ht and RLS-Ht varied with respect to the developmental stage of the host, showing the highest density in adult*s*. A relatively low density was observed in eggs, larvae and nymphs and the relative stable of density among these developmental stages potentially due to the bottleneck effect during vertical transmission [39]. A similar phenomenon was also observed in the intracellular symbiont “*Candidatus* Midichloria mitochondrii” harbored in *Ixodes ricinus* and numerous insect symbionts [40–42]. The narrow bottleneck effect could give rise to more genetic drift in symbiont populations, which would cause further genome erosion and streamlining [43, 44], and increasing evidences have been found in reduced genome of *Coxiella*-like symbionts in *A. americanum* [21] and *R. turanicus* [22].

A sex-specific distribution of CLS-Ht, with high density in females and low density in males was observed in the tick *H. tibetensis*. Similar results were also observed in the distribution of *Coxiella*-like endosymbionts in *H. hystricis*, *H. lagrangei*, *H. obesa*, *H. shimoga* [45] and *R. turanicus* [18]. However, no sex-specific distribution was observed for RLS-Ht in *H. tibetensis*. After feeding of larvae and nymphs, the density of RLS-Ht was increased when compared to the unfed group. Similar results were observed in the amount of “*Candidatus* Midichloria mitochondrii” in *I. ricinus*, hence, they were putatively involved in the feeding process of ticks [46]. Both CLS-Ht and RLS-Ht were mainly distributed in the ovaries in this study similar to the previous findings for *D. silvarum* [47]; however, the dynamics of both endosymbionts was different. The CLS-Ht density reached a peak one day after engorgement but subsequently decreased to lower levels, whereas RLS-Ht densities varied randomly while remaining relatively high in ovaries compared to other organs in females.

## Conclusions

Taken together, two novel endosymbionts (CLS-Ht and RLS-Ht) were characterized, both showing a high prevalence and stable vertical transmission in *H. tibetensis*. The described tissue distribution and population dynamics might imply the important functions of these symbionts during the development and reproduction of ticks. Further investigations are required to explore the interactions between CLS-Ht, RLS-Ht and ticks in order to further characterize the effects of the host-pathogen interactions.

## Additional files

Additional file 1: Figure S1. PCR analysis of the prevalence of CLS-Ht and RLS-Ht in *H. tibetensis* adults. (PPTX 215 kb)

Additional file 2: Figure S2. PCR analysis of the vertical transmission of CLS-Ht and RLS-Ht in *H. tibetensis. (PPTX 68 kb)*

Additional file 3: Figure S3. Detection of infection sites of two symbionts by PCR from different tissues of *H. tibetensis. (PPTX 66 kb)*

## Abbreviations

CLS-Ht: *Coxiella*-like symbiont in *H. tibetensis*
F1: The first generation
RFLP: Restriction fragment length polymorphism
RLS-Ht: *Rickettsia*-like symbiont in *H. tibetensis*
RT-qPCR: Real-time quantitative polymerase chain reaction
