# Supplementary material for: Symbiont dynamics of the Tibetan tick Haemaphysalis tibetensis (Acari: Ixodidae)
Source: Parasit Vectors. 2017 May 25;10:259. doi: 10.1186/s13071-017-2199-0 (PMC5445347; doi:10.1186/s13071-017-2199-0)
Supplement: Supplementary file 1 — PCR analysis of the prevalence of CLS-Ht and RLS-Ht in H. tibetensis adults. (PPTX 215 kb) [file 13071_2017_2199_MOESM1_ESM.pptx]

## Slide 1
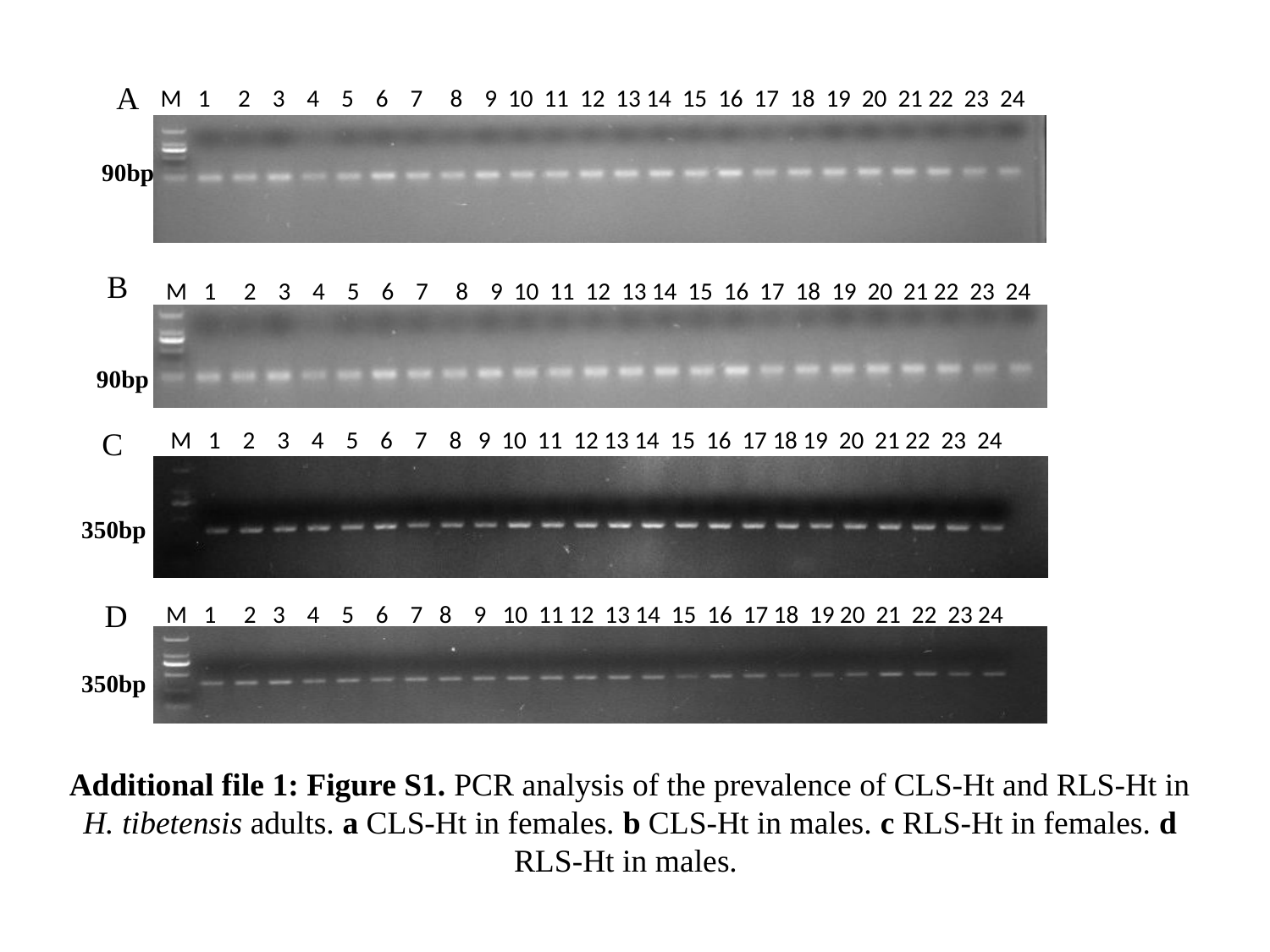

A
M 1 2 3 4 5 6 7 8 9 10 11 12 13 14 15 16 17 18 19 20 21 22 23 24
90bp
B
M 1 2 3 4 5 6 7 8 9 10 11 12 13 14 15 16 17 18 19 20 21 22 23 24
90bp
C
M 1 2 3 4 5 6 7 8 9 10 11 12 13 14 15 16 17 18 19 20 21 22 23 24
350bp
D
M 1 2 3 4 5 6 7 8 9 10 11 12 13 14 15 16 17 18 19 20 21 22 23 24
350bp
Additional file 1: Figure S1. PCR analysis of the prevalence of CLS-Ht and RLS-Ht in H. tibetensis adults. a CLS-Ht in females. b CLS-Ht in males. c RLS-Ht in females. d RLS-Ht in males.
