# Supplementary material for: Symbiont dynamics of the Tibetan tick Haemaphysalis tibetensis (Acari: Ixodidae)
Source: Parasit Vectors. 2017 May 25;10:259. doi: 10.1186/s13071-017-2199-0 (PMC5445347; doi:10.1186/s13071-017-2199-0)
Supplement: Supplementary file 2 — PCR analysis of the vertical transmission of CLS-Ht and RLS-Ht in H. tibetensis. (PPTX 68 kb) [file 13071_2017_2199_MOESM2_ESM.pptx]

## Slide 1
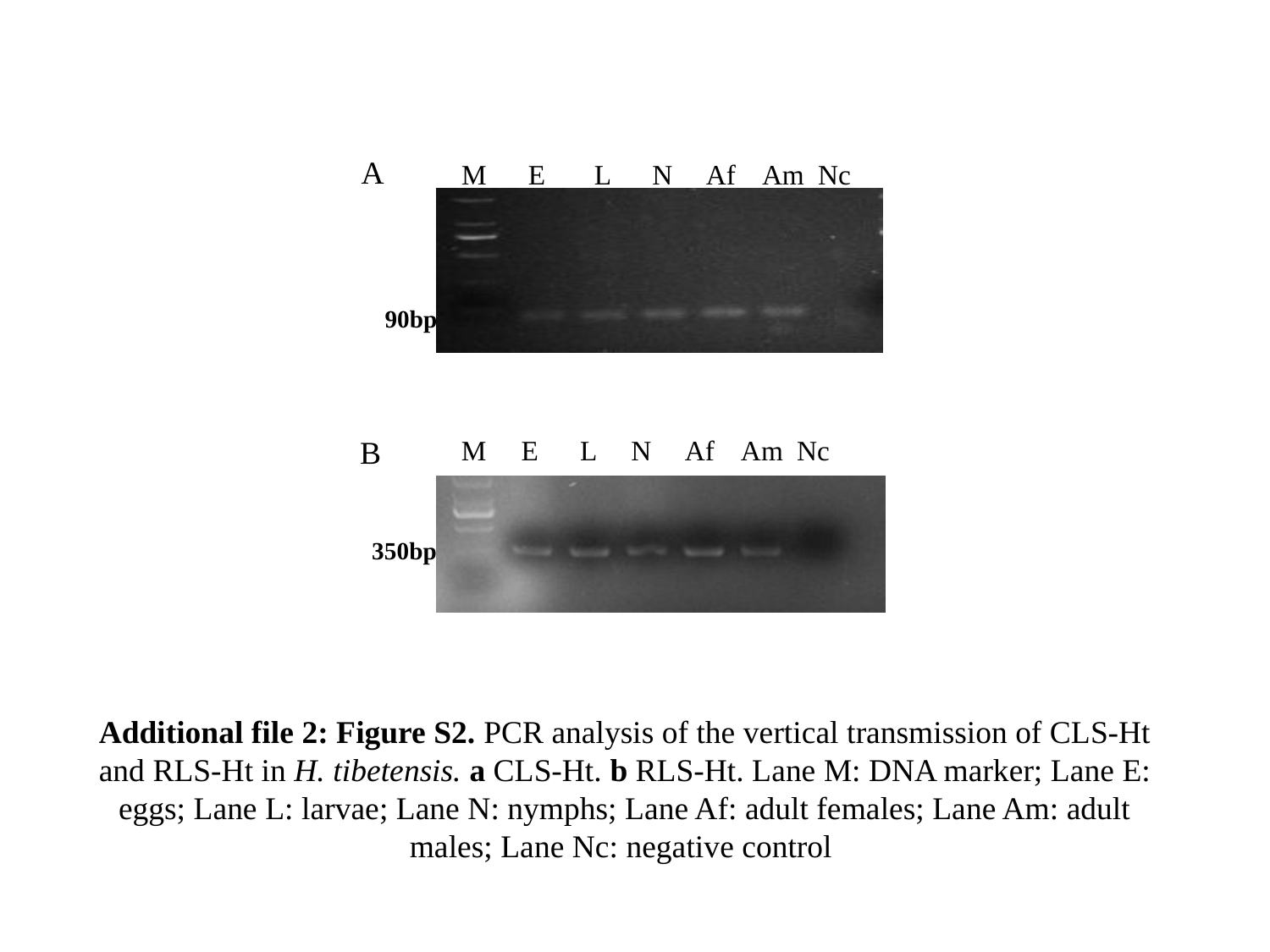

A
 M E L N Af Am Nc
90bp
B
 M E L N Af Am Nc
350bp
Additional file 2: Figure S2. PCR analysis of the vertical transmission of CLS-Ht and RLS-Ht in H. tibetensis. a CLS-Ht. b RLS-Ht. Lane M: DNA marker; Lane E: eggs; Lane L: larvae; Lane N: nymphs; Lane Af: adult females; Lane Am: adult males; Lane Nc: negative control
