# Supplementary material for: Symbiont dynamics of the Tibetan tick Haemaphysalis tibetensis (Acari: Ixodidae)
Source: Parasit Vectors. 2017 May 25;10:259. doi: 10.1186/s13071-017-2199-0 (PMC5445347; doi:10.1186/s13071-017-2199-0)
Supplement: Supplementary file 3 — Detection of infection sites of two symbionts by PCR from different tissues of H. tibetensis. (PPTX 66 kb) [file 13071_2017_2199_MOESM3_ESM.pptx]

## Slide 1
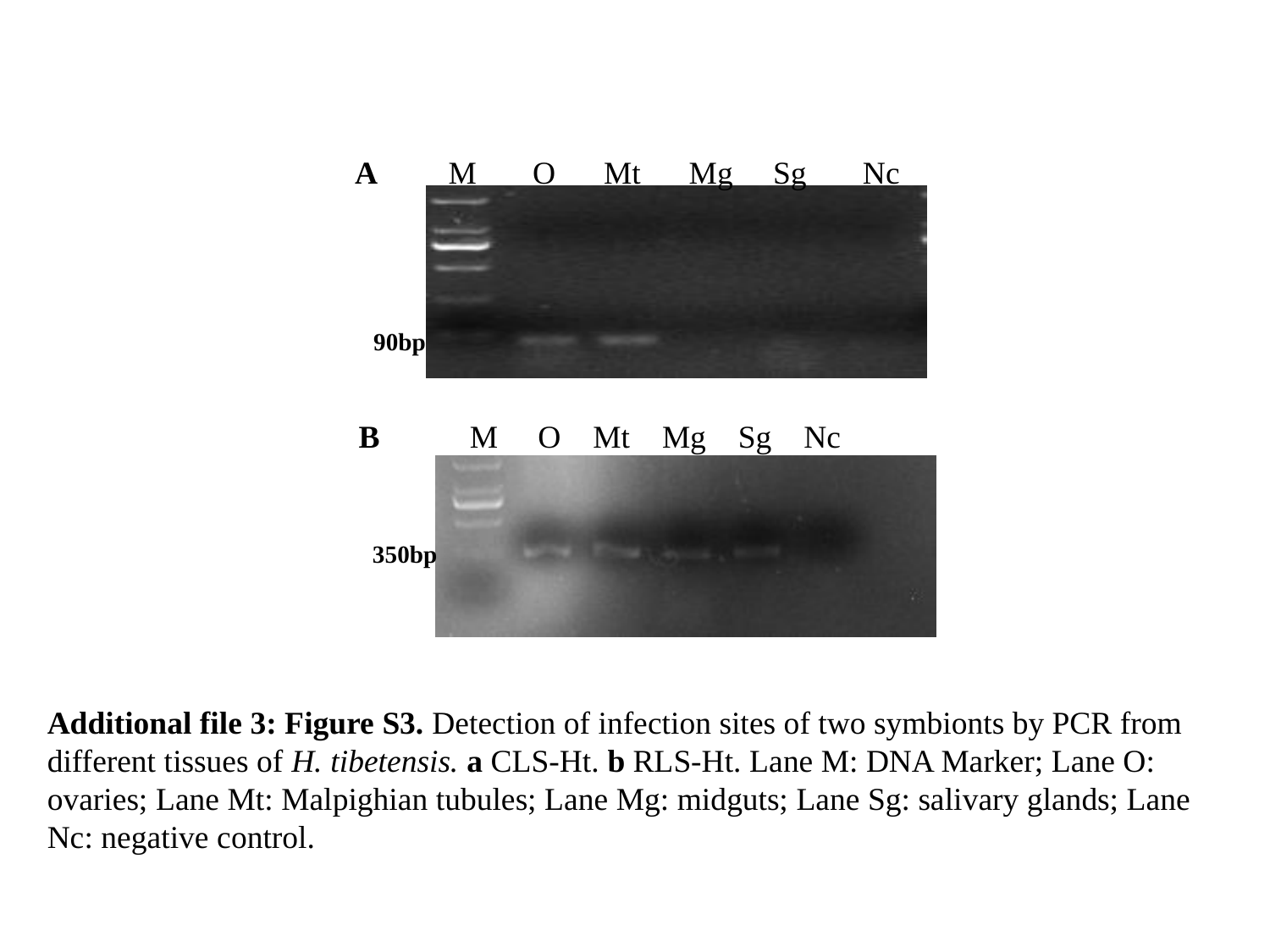

A
M O Mt Mg Sg Nc
90bp
B
M O Mt Mg Sg Nc
350bp
Additional file 3: Figure S3. Detection of infection sites of two symbionts by PCR from different tissues of H. tibetensis. a CLS-Ht. b RLS-Ht. Lane M: DNA Marker; Lane O: ovaries; Lane Mt: Malpighian tubules; Lane Mg: midguts; Lane Sg: salivary glands; Lane Nc: negative control.
